# Rapid Determination of Wine Grape Maturity Level from pH, Titratable Acidity, and Sugar Content Using Non-Destructive In Situ Infrared Spectroscopy and Multi-Head Attention Convolutional Neural Networks

**DOI:** 10.3390/s23239536

**Published:** 2023-11-30

**Authors:** Eleni Kalopesa, Theodoros Gkrimpizis, Nikiforos Samarinas, Nikolaos L. Tsakiridis, George C. Zalidis

**Affiliations:** 1 Laboratory of Remote Sensing, Spectroscopy, and GIS, School of Agriculture, Aristotle University of Thessaloniki, 57001 Thermi, Greece; gkrimpiz@agro.auth.gr (T.G.); smnikiforos@topo.auth.gr (N.S.); tsakirin@ece.auth.gr (N.L.T.); zalidis@agro.auth.gr (G.C.Z.); 2 Interbalkan Environment Center, 18 Loutron Str., 57200 Lagadas, Greece

**Keywords:** TSS, vis–NIR, NIR spectroscopy, cultivar, vineyard, deep learning, oenological parameters, total acidity

## Abstract

In the pursuit of enhancing the wine production process through the utilization of new technologies in viticulture, this study presents a novel approach for the rapid assessment of wine grape maturity levels using non-destructive, in situ infrared spectroscopy and artificial intelligence techniques. Building upon our previous work focused on estimating sugar content (${}^{\circ }$Brix) from the visible and near-infrared (VNIR) and short-wave infrared (SWIR) regions, this research expands its scope to encompass pH and titratable acidity, critical parameters determining the grape maturity degree, and in turn, wine quality, offering a more representative estimation pathway. Data were collected from four grape varieties—Chardonnay, Malagouzia, Sauvignon Blanc, and Syrah—during the 2023 harvest and pre-harvest phenological stages in the vineyards of Ktima Gerovassiliou, northern Greece. A comprehensive spectral library was developed, covering the VNIR–SWIR spectrum (350–2500 nm), with measurements performed in situ. Ground truth data for pH, titratable acidity, and sugar content were obtained using conventional laboratory methods: total soluble solids (TSS) (${}^{\circ }$Brix) by refractometry, titratable acidity by titration (expressed as mg tartaric acid per liter of must) and pH by a pH meter, analyzed at different maturation stages in the must samples. The maturity indicators were predicted from the point hyperspectral data by employing machine learning algorithms, including Partial Least Squares regression (PLS), Random Forest regression (RF), Support Vector Regression (SVR), and Convolutional Neural Networks (CNN), in conjunction with various pre-processing techniques. Multi-output models were also considered to simultaneously predict all three indicators to exploit their intercorrelations. A novel multi-input–multi-output CNN model was also proposed, incorporating a multi-head attention mechanism and enabling the identification of the spectral regions it focuses on, and thus having a higher interpretability degree. Our results indicate high accuracy in the estimation of sugar content, pH, and titratable acidity, with the best models yielding mean $R^{2}$ values of 0.84, 0.76, and 0.79, respectively, across all properties. The multi-output models did not improve the prediction results compared to the best single-output models, and the proposed CNN model was on par with the next best model. The interpretability analysis highlighted that the CNN model focused on spectral regions associated with the presence of sugars (i.e., glucose and fructose) and of the carboxylic acid group. This study underscores the potential of portable spectrometry for real-time, non-destructive assessments of wine grape maturity, thereby providing valuable tools for informed decision making in the wine production industry. By integrating pH and titratable acidity into the analysis, our approach offers a holistic view of grape quality, facilitating more comprehensive and efficient viticultural practices.

## 1. Introduction

The vine is a widely cultivated plant, and the global area under vines, which corresponds to the total area planted with vines for all uses (wine, table grapes, and raisins), including young vines not yet in production, is estimated at 7.3 million hectares in 2022. The International Organization of Vine and Wine (OIV) estimates that 258 million hectoliters of wine were produced in 2022, of which 161 million hectoliters were produced in Europe [1]. In order to avoid further interventions during vinification and to make wines with optimal organoleptic characteristics, the grapes must be harvested at their optimal maturity [2], given that grapes are non-climacteric fruits, meaning that they do not continue to ripen once harvested. Making a decision regarding when to harvest grapes at their ideal ripeness necessitates a thorough understanding of grape composition factors that are essential for achieving a desired wine style. This decision should take into account various factors such as the grape variety and the desired organoleptic characteristics of the wine, local climate [3], topography [4], seasonal weather conditions, and vineyard management practices [5]. Conventional methods employed to assess grape ripeness involve measuring the concentration of sugar in the juice as an indicator of grape sugar accumulation or monitoring changes in grape acidity through titratable acids or pH levels [6]. This requires the application of various sampling procedures during ripening, the employment of specialized staff, and the application of chemical analyses in the laboratory, which are very costly and time-consuming. Therefore, the assessment of grape maturity by accurate, contactless, non-destructive methods is desirable especially if these could be carried out automatically by an agribot [7,8].

The visible and near-infrared (VNIR, 350–1000 nm, often referred to also as Vis–NIR) and the short-wave infrared (SWIR, 1000–2500 nm) regions of the electromagnetic spectrum have been recognized as critical ranges in grape production offering valuable spectral information for the estimation of different ripening parameters and phenolic composition [9,10]. While VNIR–SWIR spectroscopy enables the detection of molecular absorptions, including overtones of fundamental absorptions in the mid-infrared and combination bands thereof [11], it is the analysis of these signals using advanced statistical methods (including machine learning and artificial intelligence techniques) that allows the quantification of indicators from the recorded spectra [12,13].

In the same context, subsets of the VNIR–SWIR range may also provide important features for ripeness estimation including the most critical ones, i.e., sugar content, pH, and titratable acidity (TA, also called total acidity); for example, the 1100–2300 nm range has been employed to estimate these and other grape quality parameters including the total phenolic content, total flavonoids, total anthocyanins, and total tannins [14]. Along the same line, Daniels et al. (2019) [15] explored the use of NIR spectroscopy to quantify total soluble solids (TSS), TA, TSS/TA, and pH non-destructively on intact bunches, achieving promising results using the Partial Least Squares (PLS) algorithm. Ping et al. (2023) [16], by using the VNIR spectra, estimated the soluble solid content (SSC) and TA of the grapes, also recording the changes in the chemical composition at different maturity levels. The Vis, NIR, and SWIR spectroscopy was also explored by Meja-Correal et al. (2023) [17] for grape TSS estimation by applying a PLS regression model and selecting the best spectral range to avoid complex and potentially overfitted regression models, concluding that the most suitable spectral range for TSS predictions was the NIR range (701–1000 nm).

In the realm of artificial intelligence (AI), attention layers [18], a fundamental component in the realm of deep learning, have witnessed significant adoption and innovation in recent years [19], particularly in the context of natural language processing and computer vision tasks [20]. These layers, often integrated within transformer architectures [21], serve to capture intricate dependencies and relationships between input elements by assigning varying degrees of relevance to different parts of the input sequence. Multi-head attention mechanisms, an extension of attention layers, allow for the simultaneous consideration of multiple sets of attention weights, enabling the model to capture a spectrum of contextual information and multiple levels of abstraction [21]. In the domain of computer vision, the fusion of multi-head attention with Convolutional Neural Networks (CNNs) has demonstrated remarkable performance in tasks such as image captioning and object detection [22]. This combination leverages the spatial hierarchy captured by CNNs with the capacity of multi-head attention to model long-range dependencies, enhancing the model’s ability to comprehend complex visual scenes and exhibit state-of-the-art performance in a multitude of image-related tasks. Furthermore, the integration of multi-head attention with CNNs contributes to the burgeoning field of explainable AI [23]. The attention mechanisms provide an inherent form of interpretability by revealing which parts of an image or input sequence the model focuses on when making predictions. This transparency can facilitate insights into the model’s decision-making process, aiding in the understanding and trustworthiness of the AI system, and thus enhancing its explainability and interpretability for both research and practical applications.

Another key concept in artificial intelligence is the use of multi-output models which can simultaneously predict multiple targets from a given set of input features and patterns [24,25]. This differentiates them from classical single-output models that in traditional supervised learning learn a function that maps each of the inputs to a single corresponding output value. A closely related concept is multi-task learning, which involves training a model to perform multiple tasks simultaneously—the key difference between the two is that in multi-task learning, diverse tasks can be trained using distinct training sets or features, whereas in multi-output learning, the output variables typically share the same training data or features [26]. In principle, the advantage of multi-output regression lies in its capacity to predict multiple correlated outputs simultaneously, enabling the model to leverage interrelations between the outputs, which enhances overall predictive accuracy and captures complex dependencies within the data.

This research builds upon our previous work and specifically the results presented in [27], where only sugar content was determined in four different grape varieties using a CNN which outperformed standard machine learning algorithms, using data collected during the harvest and pre-harvest seasons of 2020 and 2021. In the summer of 2023, new data were collected from the same grapevines and two additional maturity indicators were determined in the laboratory, namely, pH and TA.

In this paper, the main research objectives are threefold: (1) expand the local grape spectral library to further include pH and titratable acidity maturity indicators; (2) validate the performance of the pre-trained CNN model that predicts ${}^{\circ }$Brix from the in situ point spectra which were developed from past growing seasons (i.e., 2020 and 2021) [27], using data from a completely new season (2023); (3) develop new models that further predict pH and total acidity, including examining the potential use of multi-output models that simultaneously predict all three maturity parameters. Additionally, the main novelties of the paper are the following: (1) Most studies focus on laboratory-collected spectra, whereas in this paper we focus exclusively in spectra collected in situ; (2) No studies have examined the use of multi-output models to simultaneously predict multiple oenological properties simultaneously; (3) A novel multi-input–multi-output CNN that incorporates a multi-head attention mechanism is proposed, combining the information of multiple spectral pre-treatments and predicting all maturity indicators simultaneously; the multi-head attention mechanism provides the model with the capacity to examine those wavelengths which are considered most important.

## 2. Materials and Methods

### 2.1. Materials

#### 2.1.1. Study Area Description

Ktima Gerovassiliou stands as one of the foremost wine producers in Greece, nurturing an array of white and red grape varieties. This privately owned vineyard sprawls across 72 hectares, encompassing approximately 400,000 grapevines. Nestled on the slopes of Epanomi in northern Greece, it rests 25 km southeast of Thessaloniki (coordinates: 40${}^{\circ }$27${}^{\prime }$04${}^{\prime \prime }$ N, 22${}^{\circ }$55${}^{\prime }$23${}^{\prime \prime }$ E). The climate in Epanomi region exhibits a Mediterranean character (Csa according to the Köppen–Geiger scale), marked by mild winters and hot and relatively dry summers, that is tempered by refreshing sea breezes. The soil composition primarily comprises sandy textures, interspersed with occasional clay substrates and calcareous rocks. Abundant in marine fossils, the soil bears witness to its history, as the surrounding hilly terrain originated from ancient seabed deposits.

#### 2.1.2. Grape Maturity Indicators

The three grape maturity indicators studied, namely, TSS (also referred to as sugar content), pH, and TA, are affected by the presence of different molecules. The predominant sugars found in grapes and musts are glucose and fructose, while sucrose is less frequently found in most varieties. Glucose and fructose were to found to have the highest concentration with a range from 45.86 to 122.89 mg/mL and 47.64 to 131.04 mg/mL, respectively, in 98 varieties [28]. Most of the varieties of *Vitis vinifera* L. wine grapes have a glucose/fructose ratio ranging from 0.56 to 1.08 at maturity. In terms of TA, the dominant acids in grapes are tartaric acid, malic acid, and citric acid. In addition to the three basic acids, there are also phenolic acids such as coumaric acid which occur in much lower concentrations [29]. The definition of pH is more theoretical and is defined mathematically as the logarithmic index of ten of the concentration of hydroxide ions in an electrically conductive solution such as must or wine.

#### 2.1.3. Overview of the Data Collected

In our study, we investigated the following varieties of *Vitis vinifera* L.: Chardonnay, Malagouzia, Sauvignon Blanc, and Syrah. We selected these varieties with careful consideration. Malagouzia is a native Greek variety, while the other varieties are highly recognized and grown in most wine regions worldwide [1]. The vines used in the experiment were 6 years old (in 2020) and were trained on a vertical trellis with three fixed wires and pruned in a two-sided cordon system with a standard of 16 nodes per vine, while drip irrigation was chosen as the irrigation method.

In 2020 and 2021, we collected 743 samples to estimate total soluble solids (${}^{\circ }$Brix) from all four varieties. No samples were collected from the Malagouzia variety in 2020; however, twice as many samples (179 samples) were collected in 2021 compared to the other varieties. These were the data also reported in [27]. In 2023, 7 field visits were carried out (from July to September) and we revisited the same grapevines; however, the sampling process was slightly altered. Whereas in 2020–2021 single berries were sampled, in 2023 it was necessary to sample whole bunches in order to have enough grape must for laboratory analysis (approx. 750 gr). In total, 260 bunches were collected for pH and titratable acidity (TA) evaluations in addition to ${}^{\circ }$Brix evaluations to encompass all three grape-ripening parameters.

In 2020–2021, we collected in situ point hyperspectral signatures (VNIR–SWIR) from a single berry, which was subsequently crushed to ascertain its sugar content using a portable refractometer [27]. However, in 2023, the in situ point hyperspectral signatures (VNIR–SWIR) were taken from three different berries on the cluster (bunch). The grapes were collected in a plastic labelled bag and carefully placed in a cooler during transport from the field to the laboratory. Grape analyses (pH, ${}^{\circ }$Brix, and TA) were carried out in an accredited grape analysis laboratory using the methods defined by OIV, which determined these ripening parameters for each bunch.

**Figure 1:**
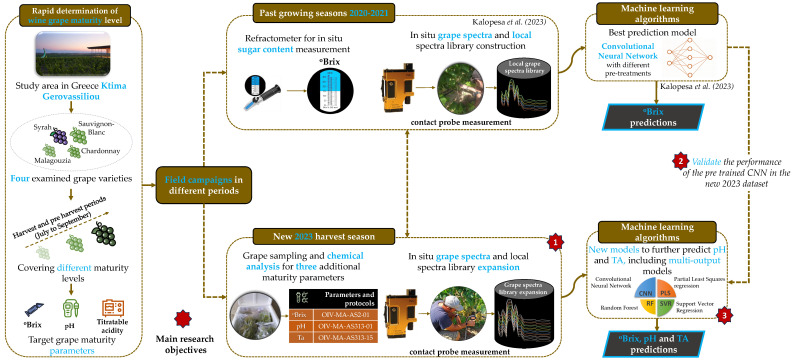
High-level overview of the applied methodology. The newly collected data reported herein were sampled in summer of 2023, while the 2020–2021 dataset and the CNN model developed from it are as reported in [27].

The overall approach is visualized in Figure 1, which both showcases the materials used and provides a high-level overview of the methods that were applied to address the three specific objectives outlined in the Introduction.

### 2.2. Methods

#### 2.2.1. Analysis of Maturity Indicators in the Laboratory

For the 2023 data, whole bunches were sampled and were analyzed in the laboratory. With respect to the sugar content, this was determined using a refractometer (OIV-MA-AS2-02). The titratable acid content was determined by using blue titration of bromothymol as an indicator and by comparison with an end-point color standard (OIV-MA-AS313-01). The pH was measured with a pH meter with a pH scale calibrated in pH units and with the sample temperature at 20 ${}^{\circ }$C (OIV-MA-AS313-15).

#### 2.2.2. Spectral Measurements and Pre-Treatments

Spectral acquisitions involved using a portable contact probe spectrometer to capture the reflectance spectra of the grapes and followed the protocol developed in our previous study [27]. More specifically, all measurements were conducted in situ, employing the PSR+3500 spectrometer from Spectral Evolution Inc., Lawrence, MA, USA. This spectrometer covers the VNIR–SWIR spectrum (350–2500 nm) and is equipped with three separate photodiode arrays for different wavelength ranges. The first array, comprising 512 Si photodiode elements, covers the 350–1000 nm range with a 2.8 nm FWHM resolution at 700 nm. The second array, featuring 256 InGaAs detector elements, spans the 970–1910 nm range with an 8 nm FWHM resolution at 1500 nm. The third array, also equipped with 256 InGaAs photodiode elements, covers the 1900–2500 nm range with a 6 nm FWHM resolution at 2100 nm. The contact probe of the spectrometer was placed in direct contact with the grape berry within the cluster to capture its spectral data. To minimize the influence of ambient light, the measurements were taken in shaded conditions, either natural or created using artificial methods. Reflectance spectra were acquired by averaging five measurements per berry, following the necessary calibration process which involved the use of a white reference plate made from Spectralon^®^ material. This calibration procedure was repeated after every ten successful spectral measurements to ensure the sensor’s ongoing accuracy and calibration. The data output of the instrument is in intervals of 1 nm.

Spectral pre-treatments in spectroscopy play a vital role in enhancing the quality of spectral data and improving the accuracy of predictive modeling for various output properties [30]. These pre-treatments are applied to raw spectral data to mitigate noise, improve signal-to-noise ratios, and extract valuable information [31]. Pseudo-absorbance is a technique used to convert reflectance or transmittance spectra into absorbance-like values, making the data more amenable to traditional absorbance-based analysis. Standard Normal Variate (SNV) normalization is employed to correct baseline shifts and variabilities in spectral data, ensuring that the spectra are directly comparable, facilitating the detection of subtle differences. Savitzky–Golay derivatives are useful for smoothing spectral data while preserving underlying trends, thereby reducing noise and sharpening spectral features, which is particularly valuable for detecting small changes in the spectra. Continuum removal, on the other hand, enables the removal of undesired background signals, isolating and highlighting spectral features of interest. Collectively, these pre-treatments are indispensable tools in spectroscopy, allowing for more robust and accurate predictions of output properties from complex spectral data.

In this paper, we tested various pre-treatments and some combinations thereof. In total, 9 different spectral sources are considered, as follows: (1) initial reflectance data (Ref), (2) the pseudo-absorbance transformation (Abs), (3) the first derivative of the absorbance (Abs+SG1), (4) the SNV transform of the absorbance (Abs+SNV), (5) SNV after the first derivative of the absorbance (Abs+SG1+SNV), (6) the second derivative of the absorbance followed by SNV (Abs+SG2+SNV), (7) the first derivative of the reflectance (Ref+SG1), (8) SNV of reflectance (Ref+SNV), (9) the continuum removal (CR). Prior to the calculation of all spectral pre-treatments, the dataset was sub-sampled by a fact of 10; thus, 216 wavelengths (or input features) are used. The parameters for all Savitzky–Golay filters that calculated the derivatives were as follows: the polynomial order was set to 3, while the window size was set to 5 data points (corresponding to approximately 50 nm).

#### 2.2.3. Proposed AI Workflow in the Current Study

To follow up on our previous work, the following steps were considered in our workflow:

1. Predict the 2023 ${}^{\circ }$Brix content using the model developed in our previous study that considered the 2020–2021 data collection campaign.
2. Build single-output ML models using the 2023 dataset to predict (independently) the maturity indicators, namely, ${}^{\circ }$Brix, pH, and TA. Here, all spectral sources are considered as input to the models independently, and the best model per each property will be reported. This process is further described in Section 2.2.4.
3. Build multi-output models to simultaneously predict all three maturity indicators. The goal is to take advantage of the cross-correlations that exist in these indicators. Moreover, a novel multi-input and multi-output CNN model employing a multi-head attention mechanism is proposed. The multi-input refers to the simultaneous use of multiple input pre-treatments to maximize the information obtained from the various spectral sources [32,33]. The goal is to establish a model that is more robust compared to the best single-output models. The proposed CNN model is described in Section 2.2.5.

#### 2.2.4. Analysis Using Standard Machine Learning Models

**Figure 2:**
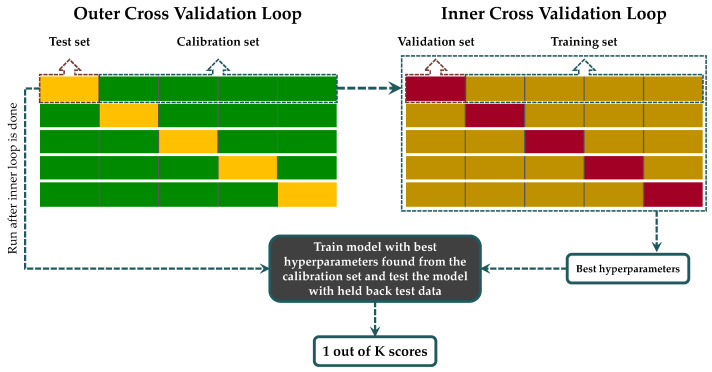
The nested cross-validation approach used in this paper. The outer loop employing in general *K* folds is used to evaluate the model’s efficacy using all available data. The inner loop, used per each iteration of the outer loop, is used to determine the optimal hyperparameters of the model. The *K* accuracy scores may be then averaged across the folds to obtain the mean accuracy scores (here, *K* = 5).

To estimate the maturity indicators of the grapes from the VNIR–SWIR spectra, different models are established per each grape variety and per each spectral pre-treatment independently. Given the relatively few samples, we opted to use a cross-validation procedure to evaluate each model’s efficacy. This is a fundamental practice in machine learning to assess the model’s performance and generalization [34]. In particular, we employed nested cross-validation using 5 folds. This process is visualized in Figure 2 and described below.

Nested cross-validation (also known as double cross-validation) combines two levels of cross-validation and unfolds in two key phases [35]. In the outer loop, the dataset is divided into five distinct folds, and the model is repeatedly trained and evaluated. Each iteration uses a different fold as an independent test set while utilizing the remaining four for model calibration. This step delivers an unbiased estimate of the model’s performance on unseen data, enabling a robust assessment of its generalization capabilities. For the outer loop, the data were split using stratified 5-fold cross-validation, with the sampling dates being the strata, thus ensuring that all our experiments considered (whether involving single-output or multi-output models) used the exact same folds.

In the inner loop, the process narrows down to fine-tuning the model’s hyperparameters. Within each loop iteration, the calibration data are further subdivided into five folds, and hyperparameter optimization takes place. During each iteration, four of these subsets were used for training the machine learning model, while the fifth subset was reserved as a validation set to fine-tune hyperparameters, with the optimal combination defined as the one that minimized the mean error of prediction across the left-out sets. This critical step allows for systematic experimentation with different hyperparameter combinations using grid search, aiming to identify the configuration that yields optimal predictive accuracy. With this optimized configuration, the model is trained on the entire calibration set, subsequently applying it to the independent test set to assess its predictive accuracy and generalization performance.

The following learning algorithms were considered in the present study:

- Partial Least Squares regression (PLS) [36], a multivariate regression technique that aims to maximize the covariance between input variables and output variables, reducing data dimensionality while preserving relevant information for more effective regression modeling. PLS has only one hyperparameter, namely, the number of latent variables; to optimize it, we searched within [1, 100].
- Random Forest (RF) [37], an ensemble learning method that constructs multiple decision trees during training and combines their predictions through voting or averaging, providing a robust and accurate model while mitigating overfitting. To optimize the hyperparameters of RF, a grid search was conducted as follows. The number of trees was selected from the {50, 100, 150, 200} set while the maximum number of features to consider when looking for the best split were selected from the {“max”, “sqrt”, “log2”} set.
- Support Vector Regressor (SVR) [38], which leverages the concept of support vectors and a margin of tolerance to find a hyperplane that best fits the data points, enabling efficient regression by maximizing the margin between predicted values and the actual target values. We selected the RBF kernel and optimized its hyperparameters through a grid search by examining the following values for ϵ: {0.01, 0.025, 0.05, 0.075, 0.10, 0.15, 0.20}, while the cost C was selected from $\left\{2^{-2},2^{-1},\ldots ,2^{9}\right\}$.

Finally, it must be noted that both PLS and RF can develop multi-output models to predict more than one target variable simultaneously [34]. SVR does not possess this capacity. Therefore, in our multi-output analysis we considered only PLS and RF and followed the exact same procedure to optimize their hyperparameters.

#### 2.2.5. Analysis Using a Novel Multi-Input and Multi-Output CNN Employing a Multi-Head Attention Mechanism

The proposed novel model aims to simultaneously predict all three grape maturity indicators. It leverages a Convolutional Neural Network (CNN) with multiple layers for feature extraction from spectral data. Crucially, as input it uses simultaneously three different pre-treatments to exploit their complimentary information; these are the Ref, Abs+SNV, and Abs+SG1. These were selected as they integrate three crucial sources of information; reflectance spectra provide valuable insights into the surface properties and overall scattering effects (i.e., the albedo), while the SNV transformation minimizes the impact of multiplicative effects, and the first derivative of absorbance enhances sensitivity to fine spectral features and absorptions [39]. The model integrates a multi-head attention mechanism to effectively process these spectral data, allowing focus on different parts of the spectra simultaneously and enhancing its ability to capture subtle variations indicative of grape maturity. Following this, we employ a stack of convolutional layers, followed by max-pooling and dropout layers to capture relevant features. Finally, the model employs fully connected layers for prediction, thereby providing estimates for all three grape maturity indicators simultaneously.

We optimized the parameters of the proposed network by employing the Hyperband algorithm [40]. With respect to the multi-head attention mechanism, the number of attention heads and the size of each attention head for query and key were optimized, which in turn helps effectively capture the complex dependencies within the spectral data. Additionally, we fine-tuned the architecture of the CNN component, adjusting the number of convolutional layers, their kernel width, and the number of filters in each layer to optimize feature extraction and spatial hierarchies. The possibility to include dropout and max-pooling layers after each convolution layer was also encoded. To ensure a well-balanced network, we also optimized the number of fully connected layers and the corresponding neurons in each layer, tailoring our model to the intricacies of the task at hand. The final optimized network structure is presented in Table 1. The parameters which were not optimized by the Hyperband algorithm but rather set externally using expert opinion were as follows: (i) The Adam optimization algorithm [41], a stochastic gradient descent method, was employed with a learning rate of 0.001l; (ii) The loss function for all three outputs was the mean squared error; (iii) All activation functions were set to ReLU; (iv) An L2 kernel weight regularization was applied on all convolutional layers with a weight of 0.01.

#### 2.2.6. Evaluating the Performance of the Models

To effectively evaluate machine learning models, it is crucial to consider a holistic approach that encompasses both accuracy metrics and model interpretability. While accuracy metrics provide essential quantitative insights into a model’s performance, they do not unveil the underlying decision-making process within complex models often referred to as “black boxes”. The element of interpretability, however, serves as a vital complement to accuracy, offering a qualitative lens through which we can understand how a model arrives at its predictions. It sheds light on the inner workings of the model, helping us determine whether it has truly identified meaningful relationships between input features and output predictions. By examining the model’s interpretability, we can uncover whether the patterns it has learned align with our domain knowledge and expectations, providing a more comprehensive assessment of its utility and trustworthiness in real-world applications.

The following metrics were used to validate the models on the independent test set, i.e., between the reference values y and the predicted values $\hat{y}$. The root mean squared error (RMSE) is calculated as:

(1) $$\mathrm{RMSE}\left(y,\hat{y}\right)=\sqrt{\frac{\sum_{i=1}^{N}\left(y_{i}-{\hat{y}}_{i}\right)}{N}}$$

with *N* being the total number of patterns, ${\hat{y}}_{i}$ being the prediction for the *i*-th pattern, and $y_{i}$ being its ground truth value.

The coefficient of determination $R^{2}$ is given by:

(2) $$R^{2}\left(y,\hat{y}\right)=1-\frac{\sum_{i=1}^{N}{\left(y_{i}-{\hat{y}}_{i}\right)}^{2}}{\sum_{i=1}^{N}{\left(y_{i}-\bar{y}\right)}^{2}}$$

where $\bar{y}$ is the mean ground truth value across all *N* patterns. The best model has an $R^{2}$ of 1, and a baseline model which always predicts $\bar{y}$ will have an $R^{2}$ of 0, with negative values indicating subpar performance.

The ratio of performance to interquartile range, RPIQ, considers both the prediction error and the variability in the observed values, and it does so without relying on any assumptions regarding the distribution of those observed values. RPIQ is quantified as the interquartile range of the observed values divided by the root mean square error (RMSE) of the predictions [42]:

(3) $$\mathrm{RPIQ}\left(y,\hat{y}\right)=\frac{Q_{3}-Q_{1}}{\mathrm{RMSE}(y,\hat{y})}$$

where $Q_{1}$ is the lower quartile or the 25th percentile of the data, whereas $Q_{3}$ is the upper quartile which corresponds with the 75th percentile.

With respect to the interpretability analysis, we analyzed the proposed multi-input–multi-output CNN model by examining the multi-head attention layer that is used directly on the input data. This enables us to discern how the model allocated its attention across the different spectral wavelengths. The attention weights, in other words, quantify the relevance of various wavelengths to the predictions. The focus was thus placed on determining the relative importance of the wavelengths.

To this end, the following steps were used. First, for each experiment (i.e., variety and fold), the relative feature importance per each wavelength is calculated. This is done by multiplying the attention weight corresponding to each input channel, attention head, and key dimension by the value of the spectral data at that wavelength. This operation captures how much attention the model assigns to each wavelength, considering all the different aspects. The weighted importances are then aggregated for each wavelength to create an overall assessment of their significance. Finally, the absolute values of the weighted importances are averaged across the folds and are normalized (dividing by the maximum value) to provide a standardized measure, allowing for a comprehensive understanding of the relative importance of different wavelengths. This approach enabled us to pinpoint the wavelengths with the most substantial influence on our model’s predictions, offering valuable insights into the spectral features critical to our analysis.

#### 2.2.7. Software Used for Model Implementation and Data Analysis

All models were implemented in Python 3.10 with data analysis taking place in the same environment. Standard ML models were developed with scikit-learn v1.3.1 [43], while the CNN models used keras v2.10.0 [44]. Results were summarized with the help of the pandas package v2.1.0 [45] and visualized with pandas and seaborn v0.12.0 [46].

## 3. Results

### 3.1. Data Collected in the 2023 Growing Season

**Figure 3:**
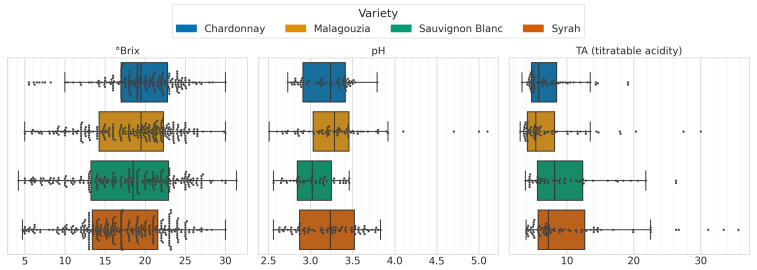
Box plot (overlaid with a swarmplot) of the ${}^{\circ }$Brix, pH, and titratable acidity content across the four different grape varieties (2023 dataset).

In this subsection, the results pertaining to the specific objective 1 noted in the Introduction are presented. The data collected and the major statistical moments thereof across the three years (2020, 2021, and 2023) are summarized in Table 2. The results of 2020–2021 were reported in [27], but are also presented in this table to aid the comparisons between the two campaigns (merged 2020–2021 campaign and 2023). To aid the comprehensibility of this table, the boxplots (overlaid with swarmplots) are also given in Figure 3, and visually present the distributions per each variety only for the 2023 data. Grape samples were collected at different stages during ripening, so that the sample set included the widest possible range of values for ${}^{\circ }$Brix, TA, and pH. Therefore, we observe that in Malagouzia there are some high values of pH out of limits as well as titratable acidity in Malagouzia and Syrah. This is due to the early harvesting of samples at maturity of these two varieties.

**Figure 4:**
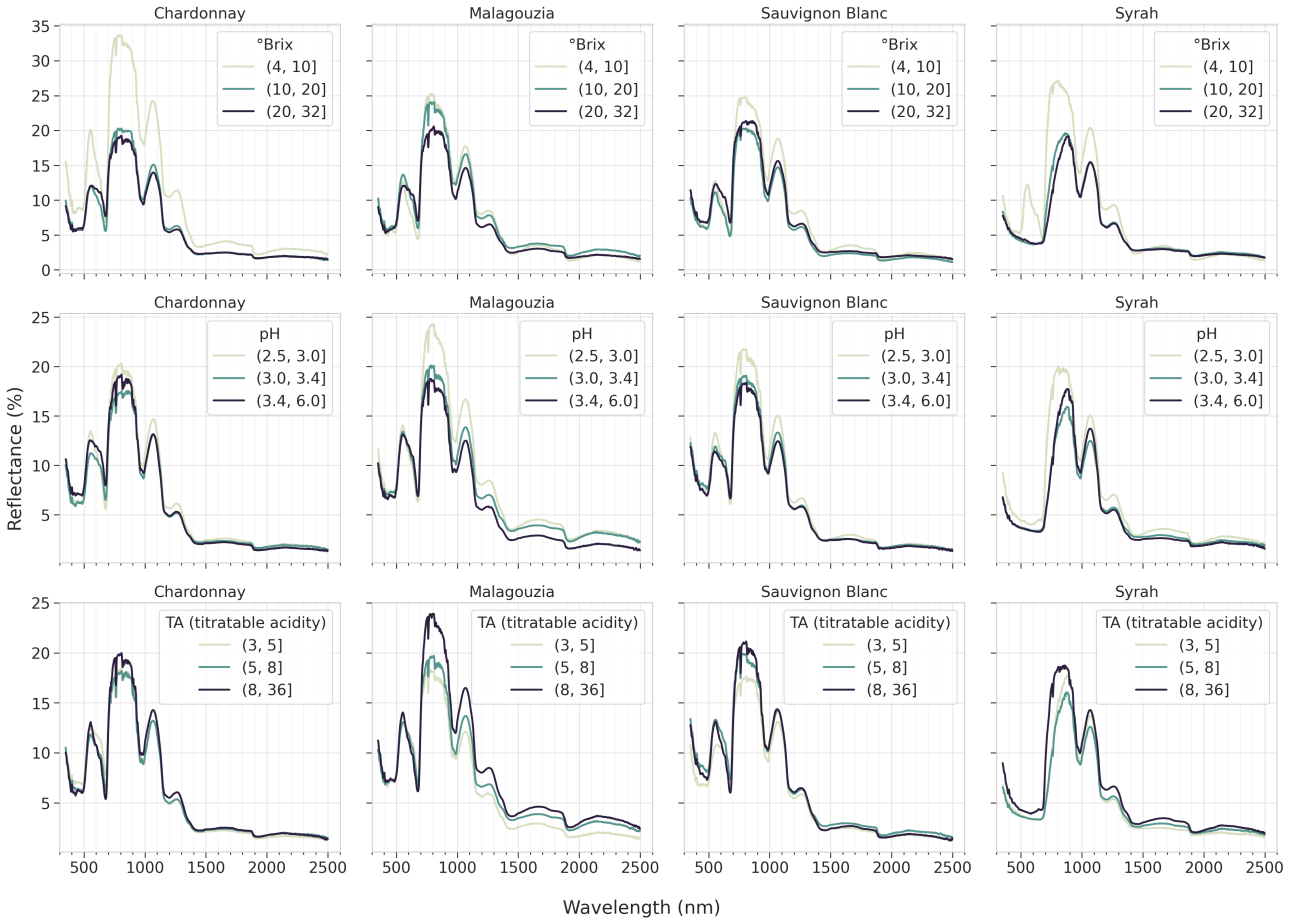
The effect each output property (grouped into three arbitrary classes, representing low, medium, and high value ranges) has on the mean reflectance spectra per each grape variety (2023 dataset).

The progress of the grapes’ reflectance as the maturity progresses is depicted in Figure 4. It appears that in Chardonnay and Syrah, higher values are obtained in the ${}^{\circ }$Brix plots in the visible range, and specifically at the 500–700 nm wavelength range. This is due to the maturity stage of the grapes as sampling started before the grapes were at the stage of veraison, when the grapes have a more greenish color, which corresponds consequently to a higher albedo (reflectance) for lower ${}^{\circ }$Brix values.

**Figure 5:**
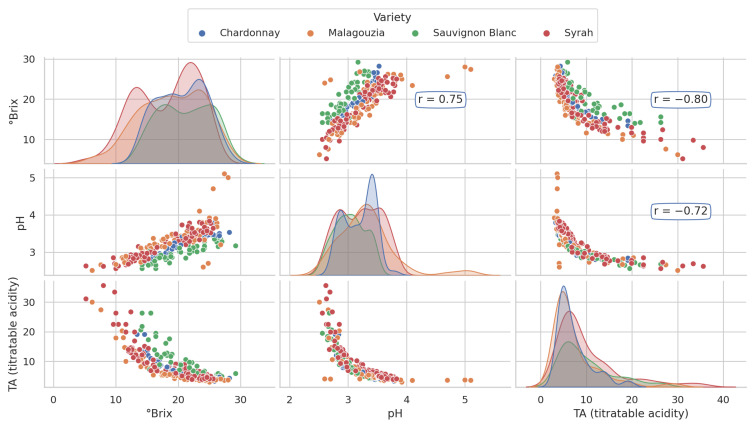
Pairplot illustrating the relationships and correlations (Pearson’s correlation denoted as r) of the oenological parameters (${}^{\circ }$Brix, pH, TA) among the four grape varieties, providing insights into their interrelations. The main diagonal displays the kernel density estimate plot per each grape variety.

The pairplot visualization of ${}^{\circ }$Brix, pH, and TA (Figure 5), with the inclusion of kernel density estimates on the main diagonal and separation by grape variety, provides a comprehensive overview of the relationships among these key parameters. The high correlations observed within this set of variables are indicative of their interconnected nature. Notably, the highest correlation is found between ${}^{\circ }$Brix (sugar content) and TA (total acidity) with a coefficient of 0.8, underlining the inverse relationship between sugar content and acidity in grapes. On the other hand, the correlation between pH and TA is slightly lower but still substantial at 0.72. This relationship emphasizes how acidity and pH levels are related, affecting the overall balance and taste of grapes.

### 3.2. Evaluation of the 2020–2021 Model on the 2023 Dataset

This section presents the performance of the CNN model developed in [27] when applied to the new dataset collected in 2023, with a comparison with the original results of the independent set from the 2020–2021 dataset (Table 3), addressing the specific objective 2. A key point that needs to be noted is that the results in 2023 are from the mean prediction across the five CNN models developed (due to the use of five-fold cross-validation). These results provide valuable insights into the model’s behavior under different varieties in a completely different cultivation year.

Most notably, the CNN model demonstrated enhanced predictive performance when applied to the 2023 dataset for Chardonnay. In 2020–2021, the model achieved an RMSE of 2.10 ${}^{\circ }$Brix, an $R^{2}$ of 0.63, and an RPIQ of 2.24. However, in 2023, these metrics improved significantly, with an RMSE of 1.96 ${}^{\circ }$Brix, $R^{2}$ of 0.74, and RPIQ of 3.39. This finding suggests that the model adapted well to the new vintage, providing more accurate predictions. In contrast to Chardonnay, the other wine varieties, Malagouzia, Sauvignon Blanc, and Syrah, experienced a decline in performance when the model was applied to the 2023 dataset. Sauvignon Blanc, in particular, exhibited a significant decrease in $R^{2}$ and RPIQ, dropping from 0.86 to 0.61 and from 4.11 to 2.48, respectively. These findings underscore the challenges of applying a model trained on historical data to new and independent datasets.

All in all, an interesting observation is the relatively consistent RMSE values across both time periods for all wine varieties. In 2020–2021, RMSE values ranged from 1.76 to 2.20, while in 2023, they ranged from 1.96 to 2.75. This consistency in RMSE values indicates that the model maintained a similar level of precision in predicting wine attributes, despite variations in $R^{2}$ and RPIQ which are affected also by the different variance and IQR values, respectively, that are found in the two datasets. Moreover, it should be noted that whereas the 2020–2021 dataset used a single berry whose sugar content was measured in situ with a portable refractometer, the 2023 dataset comprises spectra from three different berries within a single bunch whose sugar content was measured in the laboratory using conventional analytical methods.

### 3.3. Prediction of Maturity (${}^{\circ }$Brix, pH, and TA) on the 2023 Dataset

This subsection reports the results of the specific objective 3, and specifically examines the accuracy of different models (including multi-output models) on the 2023 dataset which includes all three maturity indicators. First, the results of the standard single-output machine learning models are reported, i.e., where each maturity indicator is predicted independently from the others. Then, multi-output models are examined where all maturity indicators are predicted simultaneously.

#### 3.3.1. Standard Single-Output Machine Learning Models

The results presented in Table 4 offer valuable insights into the performance of standard ML models when applied to the 2023 dataset for predicting the three critical oenological maturity indicators, namely, ${}^{\circ }$Brix, pH, and titratable acidity (TA). The table summarizes the mean performance metrics for the best combination of learning algorithm and spectral pre-treatments for each maturity indicator and grape variety.

For the prediction of ${}^{\circ }$Brix, the machine learning models generally exhibit consistent performance, with $R^{2}$ values ranging from 0.74 to 0.89 across different varieties. Chardonnay, utilizing the Random Forest (RF) model with ABS+SG1+SNV spectral pre-treatment, stands out with a notably high $R^{2}$ value of 0.86 and a high RPIQ of 4.49. This suggests that the model provides accurate and reliable predictions for the sugar content in Chardonnay grapes. Furthermore, Malagouzia with Partial Least Squares (PLS) modeling and REF spectral pre-treatment also exhibits a strong $R^{2}$ value of 0.89 and an RPIQ of 5.43, indicating good predictive performance. Overall, the machine learning models maintain consistent predictive accuracy for ${}^{\circ }$Brix, similar to the previous table discussed (Table 3).

With respect to the pH predictions, the models perform reasonably well, with Chardonnay, utilizing the RF model with ABS+SG1+SNV spectral pre-treatment, achieving a remarkable $R^{2}$ value of 0.88 and a high RPIQ of 5.72. This suggests that the model excels in predicting the acidity levels of Chardonnay grapes. However, Malagouzia displays comparatively lower predictive performance for pH, with an $R^{2}$ value of 0.44, indicating a less reliable prediction for this particular maturity indicator. The remaining varieties, Sauvignon Blanc and Syrah, exhibit moderate predictive performance. It is worth noting that, overall, the machine learning models offer satisfactory pH predictions, except for Malagouzia where improvements might be needed.

In the case of titratable acidity, the machine learning models consistently perform well. It should be noted that in all cases the best learning algorithm was RF. The models exhibit $R^{2}$ values ranging from 0.67 to 0.83, indicating good predictive accuracy for TA. Notably, Chardonnay with RF and ABS+SG1+SNV pre-treatment achieves an $R^{2}$ value of 0.67 and an RPIQ of 1.90, suggesting reliable predictions for titratable acidity. The other varieties also perform adequately well, with higher $R^{2}$ values but also higher RMSE values; this is due to their higher variability considering that the dataset includes samples that had high TA (not yet mature).

#### 3.3.2. Multi-Output Models

**Figure 6:**
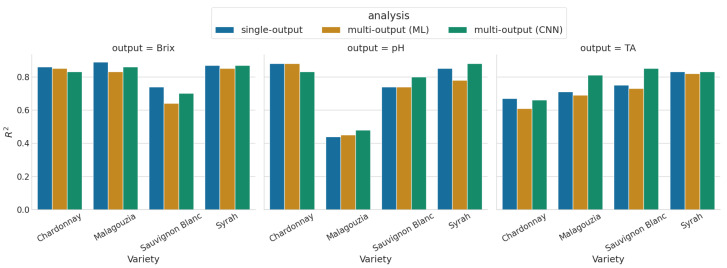
Comparison of $R^{2}$ (higher is better) between the single-output approaches (reported in Table 4) and the multi-output approaches (Table 5).

The results of the multiple output models considered are reported in Table 5. Reported are the best combination of learning algorithm (PLS or RF) and spectral pre-treatments (out of the nine considered) for the standard ML models, and the results of the proposed multi-input–multi-output CNN employing the multi-head attention mechanism. The best standard ML model was selected as the one minimizing the mean $R^{2}$ across all three maturity indicators and five-folds. To aid the comparison between the single-output models and the multi-output ones, Figure 6 provides the $R^{2}$ values in barplots.

Comparing these results, it is first clear that the proposed CNN model generally outperforms the best from the standard ML multi-output models, with only two exceptions (in Chardonnay, the ${}^{\circ }$Brix and pH predictions have lower accuracy; however, TA is predicted more accurately). However, comparing the best single-output models with the multi-output models, it may be seen that there is no clear winner. In general, the multi-output models have lower accuracy when predicting the ${}^{\circ }$Brix content, but perform slightly better in the other two maturity indicators. This may be attributed to the fact that the multi-output models strive to average out the prediction errors across all three maturity indicators, and the increase in model performance in pH and TA comes only to the detriment of the ${}^{\circ }$Brix content.

### 3.4. Model Intepretability

**Figure 7:**
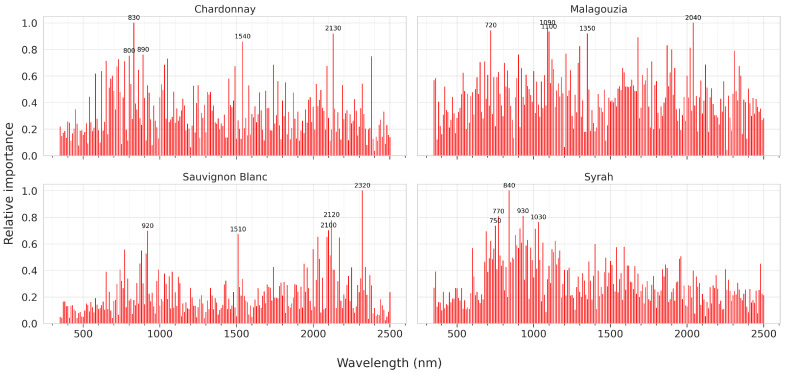
Relative feature importance per each variety across all three maturity indicators (Brix, pH, and TA) as identified by the multi-head attention layer of the proposed multi-output model.

In the interpretability analysis of the proposed model, the focus was placed on identifying the spectral regions (or features) that the multi-head attention layer deems important (Figure 7). This relative feature importance plot concerns all three maturity indicators simultaneously. Annotated on each plot are the five top-valued wavelengths for each variety.

For Chardonnay, it is evident that the focus is placed primarily between 550 and 900 nm, with distinct peaks at 640, 720, 800, 830, and 890 nm. Three more regions emerge as important, namely, around 1040, 1540, 2130, and 2390 nm. As far as Malagouzia is concerned, it is evident that the model has not performed a very sparse feature identification, with focus placed on multiple regions of the spectrum. Still, some wavelengths that emerge are at 720, 1100, 1350, and 2040 nm. The relative importance plot for Sauvignon Blanc reveals that in this cultivar SWIR is the most important spectral region. In addition to bands in VNIR at 770 and 920 nm, bands at 1510, around 2110, and at 2320 nm are where the model mostly focuses. With respect to the red wine variety (i.e., Syrah), the model tends to place particular emphasis between 550 and 1100 nm, with the top five peaks standing at 750, 770, 840, 930, and 1030 nm.

## 4. Discussion

Building on our prior research, as detailed in [27], where a CNN excelled in sugar content prediction for four grape varieties using data collected in 2020 and 2021, we present a novel approach. In the summer of 2023, new data were gathered from the same grapevines, encompassing in situ spectral measurements and laboratory determinations of pH and titratable acidity (TA). Our objectives were threefold: firstly, to enrich the local grape spectral library by incorporating pH and TA as additional maturity indicators; secondly, to validate the performance of the CNN model developed in previous seasons in predicting sugar content (${}^{\circ }$Brix) in the 2023 data; and lastly, to develop new models for pH and total acidity prediction, including an exploration of multi-output models that simultaneously predict all three maturity parameters. Practically speaking, these models may be accordingly applied to newly recorded in situ point spectra to ascertain in real time the maturity of wine grapes and thus help select the optimal harvest time. In tandem with the final point, a multi-input–multi-output CNN enhanced by a multi-head attention mechanism was proposed, which empowers the model to discern the most influential spectral wavelengths for prediction. To qualitatively describe the accuracy metrics below, “excellent fit” is used to describe $R^{2}$ above 0.8 and RPIQ above 4 while “good fit” denotes $R^{2}$ above 0.6 and RPIQ above 2.

With respect to the first goal, the grape spectral library was expanded in the 2023 field campaign to encompass pH and TA measurements, in addition to sugar content, for the four distinct grape varieties (i.e., Chardonnay, Malagouzia, Sauvignon Blanc, and Syrah), across the entire maturity period (Figure 3). The cross-correlations observed in the data (Figure 5) revealed a clear and consistent inverse relationship between acidity on the one hand and sugar content and pH on the other as the grapes ripened. Furthermore, the mean spectral plots (Figure 4) indicated significant similarities across the maturity indicators, reinforcing the hypothesis that these intercorrelations could potentially be harnessed by a single multi-output model. This insight underscores the interplay between key oenological parameters and signifies the potential for more robust and unified predictive models that leverage these intricate relationships.

As far as the second goal is concerned, there is a notable difference in data acquisition methodologies between the 2020–2021 and 2023 datasets that should be acknowledged. In the former, we employed single-berry spectral acquisitions, whereas in the latter, we adopted a more complex approach, collecting spectra from three berries within the same bunch (subsequently extracting the mean reflectance), and conducted measurements of ${}^{\circ }$Brix, pH, and TA from the must of the entire bunch after crushing. Despite this divergence in data acquisition, the results remained encouragingly consistent (Table 3). In particular, there was a relative increase in terms of RMSE of 20% (mean across the varieties), with the results for Chardonnay noting a decrease in RMSE. Across all varieties, the RPIQ metric was above 2, signifying a good fit. This discrepancy raises intriguing questions about the potential for robust predictive models that can adapt to different data collection scenarios, in addition to their usage on a completely new growing season, lending further resilience to the system.

Turning to the third objective, we developed and evaluated a range of predictive models, encompassing both single-output machine learning models (such as PLSR, RF, and SVR) and multi-output models (PLSR and RF), in addition to our novel CNN equipped with a multi-head attention mechanism. The following observations may be noted from the single-output models. First, it is evident that models developed using the 2023 data were more robust in predicting the sugar content than the best model developed from the 2020–2021 data with a mean RMSE of 1.66 ${}^{\circ }$Brix compared to 2.40, respectively (Table 4). The $R^{2}$ and RPIQ values indicate excellent fit for Chardonnay, Malagouzia, and Syrah, with Sauvignon Blanc showing good fit. Second, the prediction performance for pH and TA is mixed and changes from one variety to the other, with the red grape variety (i.e., Syrah) producing the most robust results. For example, the Chardonnay and Syrah models have excellent fit for pH, while only a good fit is observed in TA for Sauvignon Blanc and Syrah. The worst results are observed for the pH predictions of Malagouzia and for the TA predictions of Chardonnay ($R^{2}$ of 0.44 and 0.67, respectively). Third, it is important to note that the spectral source used by the best model is neither consistent through the maturity indicators nor through the different varieties. Still, the first derivative and SNV transforms emerge as the ones able to produce more robust models.

Comparing the standard multi-output models (i.e., PLSR and RF) and the proposed model with the single-output results (Figure 6), it is evident that the standard ML models in most cases perform slightly worse than their best counterparts. It is noted that the best multi-output ML model uses a single spectral source from which it predicts the maturity indicators per each variety, while crucially, the best single-output models are selected for each maturity indicator independently after considering all available spectral sources. Interestingly, it is the first and second derivatives that produce the most robust results in the standard ML multi-output models. The above indicates that despite the intercorrelations and similar trends observed, if the goal is to maximize the accuracy and apply standard ML models, then using the best combination of learning algorithm and spectral source per each maturity indicator may yield the best results. Moving to the proposed CNN model, from the same figure it is noted that the model yields slightly lower predictions in terms of sugar content, but produces better results for pH and (most notably) for TA. Overall, however, there is no clear consistent winner between the best single-output model per variety and indicator, and the proposed model. However, it should be understood that each CNN model (i.e., for each variety) is compared against the best out of 27 different models (three learning algorithms times nine spectral sources).

The outcomes of the third objective, while informative, underscored the complexity of simultaneously predicting multiple oenological properties. The multi-output models, while offering baseline performance, failed to unlock significant advantages in multi-parameter prediction. Intriguingly, the multi-output models displayed marginal improvements in predicting pH and TA, albeit at the expense of sugar content (${}^{\circ }$Brix). These findings prompt a deeper exploration of multi-output models’ utility in viticulture and oenology and highlight the challenges associated with harmonizing predictions across diverse parameters.

Compared to other studies in the literature, our results are similar or better. Fadock et al. [47], in the Syrah variety, found lower $R^{2}$ and RMSE values in predicting ${}^{\circ }$Brix, pH, and acidity, and specifically $R^{2}$ 0.70 and RMSE 1.09 for ${}^{\circ }$Brix, $R^{2}$ of 0.72 and RMSE of 0.06 for pH, and $R^{2}$ of 0.31 and RMSE of 1.25 for TA. Pampuri et al. [48] in Chardonnay reported $R^{2}$ of 0.87 and RMSE 1.90 for ${}^{\circ }$Brix, $R^{2}$ of 0.62 and RMSE of 0.14 for pH, and $R^{2}$ of 0.80 and RMSE of 3.94 for TA. Rouxinol et al. [14] also report high $R^{2}$ values for ${}^{\circ }$Brix (0.86) and TA (0.86) in red grape varieties using the 1100–2300 nm spectral range. Finally, Ping et al. [16], using the table grape variety Kyoto, reported $R^{2}$ of 0.92 and RMSE of 1.01 for ${}^{\circ }$Brix, and $R^{2}$ of 0.94 and RMSE of 1.78 for TA.

Importantly, the proposed model incorporated the multi-head attention mechanism which may be used to add an interpretability degree to the black box CNN model. Examining the relative feature importance ascribed to all maturity indicators (Figure 7), and comparing them with the findings of our previous study that only examined sugar content [27], the subsequent observations can be noted. First, the spectral region around 730 nm identified as important primarily for Malagouzia and Syrah (with noticeable peaks at the two other varieties as well) has been widely reported in the literature [49,50] and may be ascribed to the second overtone of a fundamental wide absorption band around 3390 cm${}^{-1}$ in fructose and glucose, i.e., the main dissolved solids in the aqueous solution of the grape juice, due to the O–H bonds [51]. The 1510 nm in Chardonnay and Sauvignon Blanc could be an overtone of the C=O 1730 cm${}^{-1}$ associated with the carboxylic acid group of tartaric acid [52]. In the upper SWIR, the 2120 nm identified in Chardonnay and Sauvignon Blanc may be potentially ascribed to a combination of the O–H bending and C–O stretching vibrations [53], while the 2320 nm in Sauvignon Blanc may be due to the C–O bond in glucose, and specifically the second overtone of sharp absorption bands at 1080 cm${}^{-1}$ in the fingerprint region [51]. As pH is not associated directly with specific molecules, it is difficult to present similar interpretations.

With respect to the limitations of the present study, the focus was placed only on four different varieties collected from a single estate and (for pH and TA) only within a single growing season. Notably, it is evident that there is some degree of variability in the prediction accuracy of pH and TA with respect to the variety examined. Thus, the results may not generalize well to different wine grape varieties and/or grape-growing regions and future work is necessary to ascertain their generalizability. Moreover, three berries from the whole cluster were selected to measure their VNIR–SWIR spectrum while the maturity indicators were estimated from the whole cluster. Therefore, despite our best efforts to select representative berries from the cluster, there may have been a small bias in the selection process, while the VNIR–SWIR spectrum from which the maturity indicators are estimated is only a partial snapshot of the whole cluster. This may only be ameliorated through the use of setup that employs a hyperspectral imaging camera in order to capture the spectrum of the whole cluster.

In the future, this work can be extended to include more grape varieties and data from multiple growing seasons (particularly for pH and TA). Different multi-output models may also be studied to ascertain whether the simultaneous prediction can result in enhanced accuracy of prediction. Another potential avenue of research is to examine whether the use of models that incorporate multiple varieties in the calibration set are as robust as the variety-specific models that have been developed herein. Although most studies develop independent models for each cultivar, some studies focusing on table grapes have developed a single global model [15] while others have compared the accuracy between (i) a global model and (ii) white- and red-grape-specific models [54]. The use of in situ hyperspectral imaging is also a promising research avenue to transfer the models to automated agricultural robots that can determine the maturity degree of the entire field [55].

## 5. Conclusions

This paper utilized VNIR–SWIR in situ point spectroscopy to estimate three grape maturity indicators which are essential to determine the optimal harvest time. First, the generalization ability of our previously developed CNN model predicting only the sugar content was examined by applying it to a new growing season, which indicated robust predictions despite small modifications to the measurement protocol pertaining to the use of single berries (used in previous growing seasons) and the use of multiple berries from a single bunch (used in the new season). Additionally, the predictive performance of VNIR–SWIR in situ spectroscopy was evaluated in two further maturity indicators, namely, pH and TA. Overall, pH and TA are predicted with satisfying accuracy (average $R^{2}$ of 0.76 for pH and 0.79 TA across all varieties), with the worst results noted for Malagouzia ($R^{2}$ of 0.48 for pH) and Chardonnay ($R^{2}$ of 0.67 for TA), while for the sugar content the average $R^{2}$ across all varieties is 0.84. Despite noting significant intercorrelations between the three maturity indicators, the use of multi-output models did not enhance the accuracy of performance. The proposed multi-input–multi-output CNN incorporating a multi-head attention mechanism noted the most robust results for pH and TA, albeit at the cost of slightly reduced accuracy for sugar content. The multi-head attention layer was incorporated to lend an interpretability degree to the black box CNN model in order to identify the spectral regions it focuses on, which were noted to have physical interpretations with various molecular bonds.

## Figures and Tables

**Table 1:** The architecture of the proposed multi-input–multi-output CNN to simultaneously predict all three grape maturity indicators (Brix, pH, and TA); the multi-head attention mechanism focuses on different parts of the spectrum. *L* is the length of each spectrum (here 216) and *D* refers to the dimension of the multiple inputs (here 3).

Layer	Output Shape	Description
Input (Spectral Data)	(L,D)	Input layer for VNIR–SWIR spectra
Multi-Head Attention	(None, L,D)	Multi-head attention mechanism (8 heads of size 64)
Conv1D	(None, *L*, 128)	Convolutional layer with 128 filters and kernel size 5
Dropout	(None, *L*, 128)	Dropout layer with rate 0.2
MaxPooling1D	(None, L/2, 128)	Max-pooling layer with pool size 2
Conv1D	(None, L/2, 64)	Convolutional layer with 32 filters and kernel size 7
Dropout	(None, L/2, 64)	Dropout layer with rate 0.2
MaxPooling1D	(None, L/4, 64)	Max-pooling layer with pool size 2
Conv1D	(None, L/4, 32)	Convolutional layer with 16 filters and kernel size 7
Flatten	(None, L/4×32)	Flatten layer
Dense	(None, 128)	Fully connected layer, 64 units and ReLU activation
Dense	(None, 64)	Fully connected layer, 64 units and ReLU activation
Dense	(None, 32)	Fully connected layer, 32 units and ReLU activation
Output (Brix)	(None, 1)	Regression output for ${}^{\circ }$Brix
Output (pH)	(None, 1)	Regression output for pH
Output (TA)	(None, 1)	Regression output for TA

**Table 2:** Major statistical moments of the ${}^{\circ }$Brix, pH, and titratable acidity content across the four different grape varieties that were collected. Std is the standard deviation, Min and Max are the minimum and maximum values, and $Q_{1}$, $Q_{2}$, and $Q_{3}$ are the 1st, 2nd, and 3rd Quartile or 25th, 50th, and 75th percentiles, respectively.

Variety	Year	N	Mean	Std.	Min	$Q_{1}$	$Q_{2}$	$Q_{3}$	Max
**${}^{\circ }$Brix**									
Chardonnay	2020	39	19.40	2.11	14.2	18.15	19.30	20.75	23.2
	2021	100	19.86	4.06	10.00	17.00	19.65	22.92	30.00
	2023	70	19.06	5.86	5.5	17.00	20.25	23.80	26.8
Malagouzia	2021	179	18.63	5.06	6.80	14.75	20.00	22.30	30.00
	2023	80	17.54	6.08	5.00	14.00	18.25	22.12	28.20
Sauvignon Blanc	2020	95	15.15	5.61	4.20	10.90	15.00	20.60	24.50
	2021	100	19.96	5.72	7.50	15.85	20.10	25.00	31.40
	2023	60	18.99	6.51	5.60	16.75	20.25	23.52	28.20
Syrah	2020	110	16.29	4.84	4.90	13.00	16.20	19.08	25.50
	2021	120	18.09	5.08	5.50	14.20	17.90	22.25	30.00
	2023	90	17.13	6.06	4.70	13.00	18.85	22.00	26.50
**pH**									
Chardonnay	2023	60	3.19	0.26	2.73	2.91	3.24	3.41	3.79
Malagouzia	2023	70	3.31	0.48	2.51	3.03	3.28	3.46	5.10
Sauvignon Blanc	2023	50	3.04	0.26	2.56	2.84	3.02	3.25	3.46
Syrah	2023	80	3.21	0.36	2.56	2.87	3.24	3.52	3.83
**Titratable acidity**									
Chardonnay	2023	60	7.25	3.67	3.3	4.7	5.8	8.48	19.1
Malagouzia	2023	70	7.46	5.34	3.0	4.10	5.35	8.15	30.0
Sauvignon Blanc	2023	50	9.93	5.84	3.8	5.60	8.15	12.38	26.3
Syrah	2023	80	10.18	7.08	3.9	5.68	7.2	12.68	35.6

**Table 3:** Results of the CNN model developed on the 2020–2021 dataset applied on the 2023 data. The model’s configuration and the 2020–2021 results are as reported in [27].

	2020–2021				2023		
**Variety**	**RMSE**	$R^{2}$	**RPIQ**		**RMSE**	$R^{2}$	**RPIQ**
Chardonnay	2.10	0.63	2.24		1.96	0.74	3.39
Malagouzia	1.96	0.84	4.18		2.75	0.71	3.02
Sauvignon Blanc	2.20	0.86	4.11		2.57	0.61	2.48
Syrah	1.76	0.87	4.26		2.32	0.78	3.73

**Table 4:** Results of the single-output models: Mean performance of the best combination of learning algorithm and spectral pre-treatments in the independent test set using 5-fold cross-validation, per each maturity indicator and variety, using the data collected in 2023.

Variety	Model	Spectral Pre-Treatment	RMSE	$R^{2}$	RPIQ
**${}^{\circ }$Brix**					
Chardonnay	RF	ABS+SG1+SNV	1.39	0.86	4.49
Malagouzia	PLS	REF	1.61	0.89	5.43
Sauvignon Blanc	PLS	ABS+SG1	1.91	0.74	3.85
Syrah	PLS	REF+SNV	1.76	0.87	4.89
**pH**					
Chardonnay	RF	ABS+SG1+SNV	0.09	0.88	5.72
Malagouzia	SVR	REF+SNV	0.35	0.44	1.36
Sauvignon Blanc	SVR	ABS+SG1	0.13	0.74	3.06
Syrah	SVR	REF+SNV	0.13	0.85	4.51
**Titratable acidity**					
Chardonnay	RF	ABS+SG1+SNV	2.06	0.67	1.90
Malagouzia	RF	REF+SG1	2.46	0.71	1.68
Sauvignon Blanc	RF	ABS+SG1	2.75	0.75	2.82
Syrah	RF	ABS+SG1+SNV	2.85	0.83	2.98

**Table 5:** Results of the multiple output models in the independent test set using five-fold cross-validation. Reported are only the best learning algorithm and spectral pre-treatment combination when considering the standard ML models.

	Standard ML Models						Proposed CNN		
**Variety**	**Model**	**Spectral Pre-Treatment**	**RMSE**	$R^{2}$	**RPIQ**		**RMSE**	$R^{2}$	**RPIQ**
**${}^{\circ }$Brix**									
Chardonnay	RF	Abs+SG1+SNV	1.44	0.85	4.34		1.54	0.83	3.96
Malagouzia	RF	Abs+SG2+SNV	2.04	0.83	3.90		1.87	0.86	4.36
Sauvignon Blanc	PLS	Abs+SG1	2.36	0.64	2.89		2.02	0.70	3.44
Syrah	RF	Abs+SG1	1.89	0.85	4.48		1.79	0.87	4.69
**pH**									
Chardonnay	RF	Abs+SG1+SNV	0.09	0.88	6.32		0.10	0.83	4.45
Malagouzia	RF	Abs+SG2+SNV	0.35	0.45	1.35		0.31	0.48	1.49
Sauvignon Blanc	PLS	Abs+SG1	0.13	0.74	2.98		0.11	0.80	3.49
Syrah	RF	Abs+SG1	0.17	0.78	3.65		0.12	0.88	4.93
**Titratable acidity**									
Chardonnay	RF	Abs+SG1+SNV	2.16	0.61	1.80		2.11	0.66	1.84
Malagouzia	RF	Abs+SG2+SNV	2.53	0.69	1.66		2.05	0.81	2.00
Sauvignon Blanc	PLS	Abs+SG1	2.95	0.73	2.22		2.14	0.85	3.05
Syrah	RF	Abs+SG1	2.92	0.82	2.92		2.76	0.83	3.25

## Data Availability

The data presented in this study are available on request from the corresponding author.

## Abbreviations

The following abbreviations are used in this manuscript:

AI	Artificial Intelligence
CNN	Convolutional Neural Network
CV	Cross-Validation
NIR	Near-Infrared
OIV	International Organization of Vine and Wine
PLS	Partial Least Squares
RF	Random Forest
SSC	Soluble Solids Content
SVR	Support Vector Regression
SWIR	Short-Wave Infrared
TA	Titratable Acidity
TSS	Total Soluble Solids
